# Modeling and mobile home monitoring of behavioral and psychological symptoms of dementia (BPSD)

**DOI:** 10.1186/s12888-024-05579-5

**Published:** 2024-03-09

**Authors:** Haihang Yuan, Tianyi Yang, Qiaolian Xie, Guilhem Lledos, Wen-Huei Chou, Wenwei Yu

**Affiliations:** 1 Department of Medical Engineering, Chiba University, Chiba, Japan; 2 Institute of Rehabilitation Engineering and Technology, University of Shanghai for Science and Technology, Shanghai, China; 3 UPSSITECH - Paul Sabatier University of Toulouse, Toulouse, France; 4 Department of Digital Media Design, National Yunlin University of Science and Technology, Yunlin, Taiwan; 5 Center for Frontier Medical Engineering, Chiba University, Chiba, Japan

**Keywords:** BPSD models, Home monitoring robots, Identification of occurrence of BPSD

## Abstract

With the increasing global aging population, dementia care has rapidly become a major social problem. Current diagnosis of Behavior and Psychological Symptoms of Dementia (BPSD) relies on clinical interviews, and behavioral rating scales based on a period of behavior observation, but these methods are not suitable for identification of occurrence of BPSD in the daily living, which is necessary for providing appropriate interventions for dementia, though, has been studied by few research groups in the literature. To address these issues, in this study developed a BPSD monitoring system consisting of a Psycho-Cognitive (PsyCo) BPSD model, a Behavior-Physio-Environment (BePhyEn) BPSD model, and an implementation platform. The PsyCo BPSD model provides BPSD assessment support to caregivers and care providers, while the BePhyEn BPSD model provides instantaneous alerts for BPSD enabled by a 24-hour home monitoring platform for early intervention, and thereby alleviation of burden to patients and caregivers. Data for acquiring the models were generated through extensive literature review and regularity determined. A mobile robot was utilized as the implementation platform for improving sensitivity of sensors for home monitoring, and elderly individual following algorithms were investigated. Experiments in a virtual home environment showed that, a virtual BPSD elderly individual can be followed safely by the robot, and BPSD occurrence could be identified accurately, demonstrating the possibility of modeling and identification of BPSD in home environment.

## 1 Introduction

With the increase of the global aging population, dementia care becomes a worldwide growing concern. It is estimated that by 2050, 132 million elderly will suffer from the disease [1, 2]. Dementia is often accompanied by many Behavioral and Psychological Symptoms of Dementia (BPSD), and studies have shown that 89.4% of people with Alzheimer’s disease, one major type of dementia, have at least one type of BPSD [3, 4]. BPSD manifests as physical aggression, screaming, depression, and delusions, etc. These behaviors can place a significant burden on caregivers and care providers [5, 6], and reduce the quality of life for both patients, caregivers, and care providers.

Many factors can affect BPSD. Some studies have shown that impaired communication is related to various forms of aggression [7]. Some studies showed that discomfort contributes significantly to the variance in overall agitation [8]. And depression has been found to be significantly correlated with wandering [9]. However, most of this research is about statistics of one or two single symptom manifestations of a single BPSD. This study aims to quantify the influence of multivariate factors to one or more BPSDs.

The current diagnosis of dementia requires clinical interviews, and behavioral rating scales based on a period of behavior observation. The specialized scales such as the Neuropsychiatric Inventory (NPI) [10], Alzheimer Disease Assessment Scale-Cognitive Test (ADAS-Cog) [11], Behavioral Pathology in Alzheimer’s Disease rating scale (BEHAVE-AD) [12], and Cohen-Mansfield Agitation Inventory (CMAI) [13], etc. However, compared to the care facility, in the home where may be a lack of professional guidance, it can be challenging to utilize such scales for assessment. If BPSD could be properly modeled and its occurrence can be identified at home, more information could be provided to caregivers and care providers to help them make more accurate identifications and reduce their care burden [14].

Models of Dementia have been studied. A computer simulation model was established to simulate the daily lives of dementia, associating memory loss with changes in activities/tasks [15]. The model of the Dynamic Biomarker [16] and the model of brain function of dementia patients [17] have been proposed. But all of them can’t be applied to BPSD. There have been few studies on BPSD models. The Multiple Indicators Multiple Causes (MIMIC) model was built for effectively capturing the complexity of the interrelationships among symptoms, factors, and clinical variables [18]. However, this model does not account for the relationship between multiple behaviors of daily living and multiple BPSD and are unsuitable for identify BPSD at home.

An analysis of 25-year literature related to Creutzfeldt-Jakob disease, a type of dementia, found that 80% of patients exhibit BPSD within the first 100 days of onset [19]. As family members may not be able to provide round-the-clock care for the elderly, as well as the fact that BPSD may not have severe manifestations, they are often overlooked, resulting in missed opportunities for early diagnosis and intervention. Therefore, a model based on daily living behaviors and physiological states which can be observed or measured in the daily living is needed for the purpose of home monitoring of BPSD.

In this study, a BPSD monitoring system consisting of two models and a monitoring platform is proposed. The first model is a Psycho-Cognitive (PsyCo) BPSD model, which establishes the relationship between some psychological tests, cognitive scales about the patient, and BPSD, for providing BPSD assessment support to caregivers and care providers. The second model is a Behavior-Physio-Environment (BePhyEn) BPSD model, which provides instantaneous alerts for BPSD enabled by a 24-hour home monitoring platform.

Data for acquiring the models were generated through extensive literature review and regularity determined, which are described in Methods section. As there are multiple types of BPSD, this study has focused on three specific symptoms: agitation, apathy, and depression. These symptoms are selected due to their prevalence and the possibility of their identification with behavior observation and physiological measurement [3, 20]. The three symptoms are described as follows:

- **Agitation**: defined as inappropriate speech, sound, or motor activity, such as jumping, repetitive movements, dressing, undressing, and so on. A state of agitation is accompanied by increased tension and irritability [21].
- **Apathy**: often accompanied by a lack of interest in daily activities and personal care, reduced cognitive activity and mood, and reduced different types of interaction. The main difference with depression is the absence of irritability [22].
- **Depression**: accompanied by feeling of inadequacy and guilty, low energy, appetite, and sleep disorder characterized by loss of sensation. The BPSD is quite closely related to the expression of fear, anxiety, tension, and panic [22].

A mobile robot was utilized as the implementation platform for improving sensitivity of sensors for home monitoring, and elderly individual following algorithms were investigated.

Considering the complexit1y and variability of home environment, the sensors placed in environment may have occlusion or dead-zone problems. The mobile robot can not only adjust the angle and position to find the best measurement location, combine multiple sensors to meet the detection requirements, but also detect the daily activities of elderly individuals [23–25]. On the other hand, many questionnaire studies show that care robots can play an important role in the field of care [26–28]. Therefore, a mobile robot is designed as the monitoring platform to follow the elderly individual, measure, and observe his/her behavioral and physiological data.

Due to the complexity and uncertainty of home environment and the diversity of target (elderly individual) walking speed and trajectories, robots may easily lose their targets. In this study, different following algorithms were compared in different virtual home environments in terms of their performance indexes described.

The use scenario of the home BPSD monitoring system is as follows: The mobile robot keeps observing and measuring the elderly individual for 24 h, does BPSD evaluation with the BePhyEn BPSD model, and gives a timely alert, if necessary, together with a short record of data logged. After receiving the alert, the family members can make use of the PsyCo BPSD model to have a basic assessment of the risk of BPSD by checking the short record and make a final identification. The potential interventions include individualized music therapy [29] and/or reminiscence therapy [30], etc., which are out of the scope of this study.

This paper is organized as follows. In Sect. 2, the design thought and model construction methods are described. In Sect. 3, the experiment design for testing the BePhyEn BPSD model and testing three following algorithms are described. And Sect. 4 shows the results of the experiment. Sect. 5 is a discussion of these results. Finally, the conclusions of this study are described in Sect. 6.

## 2 Methods

### 2.1 System overview

**Fig. 1:**
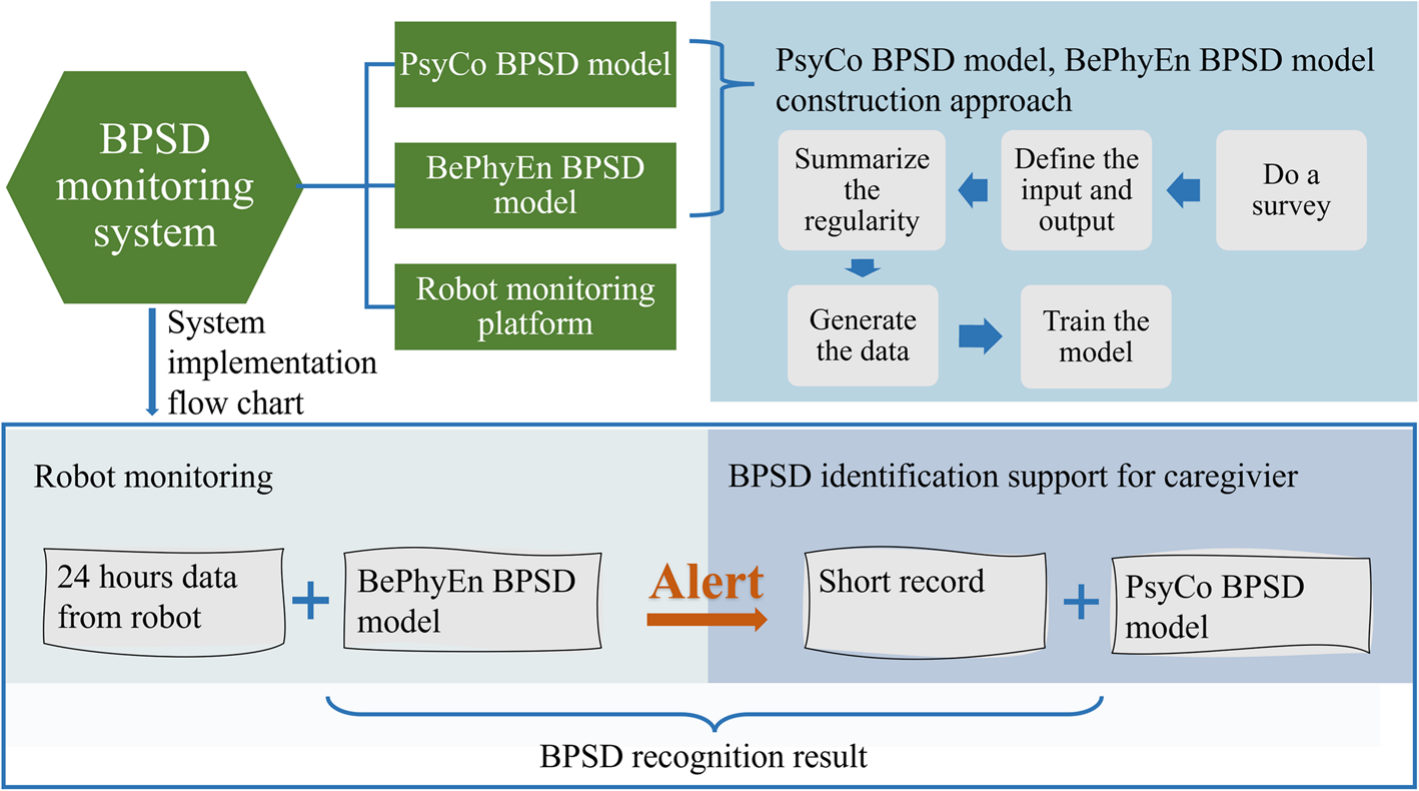
BPSD monitoring system

As shown in Fig. 1, the BPSD monitoring system consists of the PsyCo BPSD model, the BePhyEn BPSD model, and a mobile robot monitoring platform. Generally, the input to the PsyCo BPSD model can be observed and recorded by caregivers and care providers but cannot be observed or measured by sensors. The input to the BePhyEn BPSD model can be detected by sensors.

The approach to the construction of the two models is overviewed in Fig. 1, too. First, various factors such as physiological, environmental, psychological, behavioral, and other factors that affect BPSD were reviewed. These factors or manifestations were used as input to the models, while BPSD types were used as outputs. Then, based on many previous studies, the relationships between these factors and BPSD types were identified and designated as the regularities in these models. An amount of data generated by the regularities was then used to learn the models using decision tree algorithms, which can extract information structure simple to understand and to interpret, based on information theory.

### 2.2 Construction of the PsyCo BPSD model results

#### 2.2.1 To select the factors

The qualitative relationship between different factors and BPSD could be found in several databases [7, 8, 22, 31–38]. To construct the PsyCo BPSD model for the monitoring system, two criteria were considered: those reflecting the relationship between a factor and at least one of the three selected BPSDs, and those with the factor that can be observed or acquired by the caregivers and care providers. It should be point that the accuracy of the Mini-Mental State Examination (MMSE) is not high enough to give an absolute decision [31]. And it is possible to combine MMSE with other methods to identify BPSD with precision. Based on the same considerations, the other results of psychological tests, cognitive scales, are all treated as factors of PsyCo BPSD model.

For example, it has been shown that somatic diseases are associated with agitation and that more severe somatic diseases lead to more severe agitation, so somatic diseases are considered as a factor that influences agitation.

The factors selected similarly are shown in Table 1.

**Table 1:** The relationship between factors and BPSD

Factor	Agitation	Apathy	Depression
Impaired communication [7]	○		○
Somatic diseases [8]	○		
MMSE^a^ [31]	○	○	○
Weight loss [32–34]		○	
Loss of insight [36, 37]		○	○
Stressful events [22]			○
Self-esteem [22]			○
Anxiety [38]			○

○indicates that this factor affects this type of BPSD

^a^*MMSE* Mini-Mental State Examination

#### 2.2.2 To quantify the factors

As preparation of the modelling, the selected BPSDs are assigned a nominal label as follows:

- ‘0’: represents Agitation.
- ‘1’: represents Apathy.
- ‘2’: represents Depression.
- ‘3’: represents no BPSD.

In the literature of dementia research, the factors have been divided into 4 ordinal levels: 0 (no change was observed) to 3 (the drastic change was observed) [8]. In this study, impaired communication, somatic diseases, weight loss, loss of insight, stressful events, self-esteem, and anxiety are divided into four levels. Level 0 means no such situation, level 1 means low change, level 2 means medium change and level 3 means high change. Since MMSE is a widely used quantitative scale, it is kept the same. The quantitative description of the factors is summarized in Table 2.

**Table 2:** Quantitative description of factors

Factor	Quantitative definition
Impaired communication	[0 1 2 3] for [Nought Low Medium High]
Somatic diseases	
Weight loss	
Loss of insight	
Stressful events	
Self-esteem	
Anxiety	
MMSE^a^	[0 ~ 30] for [poor mental state ~ good mental state]

^a^ *MMSE* Mini-Mental State Examination

#### 2.2.3 To determine the regularity of factor level range

Since each BPSD has different severity, which affects its manifestations and levels [8, 38]. In this study, only its influence on the factor level range is considered.

Suppose that BPSD has three levels, ‘high’, ‘mild’ and ‘non’, indicating severe, intermediate and no symptoms, respectively. It is reasonable to assume that a severer BPSD has serious influence on its manifestations, i.e., factors. As an example, if an elderly individual has highly severe agitation, then it is highly likely for him/her to have severely impaired communication, more serious somatic diseases, and poorer MMSE scores. Based on this assumption, regularities for a certain BPSD are determined for modeling as follows:

For a related factor:

- high BPSD: level range (2–3).
- mild BPSD: level range (1–3).
- no BPSD: level range (0–1).

For an unrelated factor (other than MMSE and anxiety):

- high BPSD, mild BPSD, and no BPSD: level range (0–2).

For MMSE: set referring to [39].

- high BPSD: 0–22.
- mild BPSD: 22–26.
- no BPSD: 27–30.

For anxiety: set referring to [40], which denotes that anxiety level decreases as the severity of dementia increases.

- high BPSD: level range (1–3).
- mild BPSD: level range (2–3).
- no BPSD: level range (0–1).

Note that, for simplicity, the cross interaction between different BPSDs is not considered. With the determined regularities, all the levels for related and unrelated factors for the three BPSD with different severity levels (high, mild, no) are determined in Table 3.

**Table 3:** Regularities between the quantified factors and the three levels of BPSD. The items in yellow, orange, blue represent agitation, apathy and depression-related factors, respectively

	High agitation	Mild agitation	No agitation	High apathy	Mild apathy	No apathy	High depression	Mild depression	No depression
Impaired communication	2–3	1–3	0–1	0–2	0–2	0–2	2–3	1–3	0–1
Somatic diseases	2–3	1–3	0–1	0–2	0–2	0–2	0–2	0–2	0–2
MMSE^a^	0–22	22–27	27–30	0–22	22–27	27–30	0–22	22–27	27–30
Weight loss	0–2	0–2	0–2	2–3	1–3	0–1	0–2	0–2	0–2
Loss of insight	0–2	0–2	0–2	2–3	1–3	0–1	2–3	1–3	0–1
Stressful events	0–2	0–2	0–2	0–2	0–2	0–2	2–3	1–3	0–1
Self-esteem	0–2	0–2	0–2	0–2	0–2	0–2	2–3	1–3	0–1
Anxiety	0–2	0–2	0–2	0–2	0–2	0–2	1–3	2–3	0–1

^a^*MMSE* Mini-Mental State Examination

#### 2.2.4 To generate the data for training the PsyCo BPSD model

**Fig. 2:**
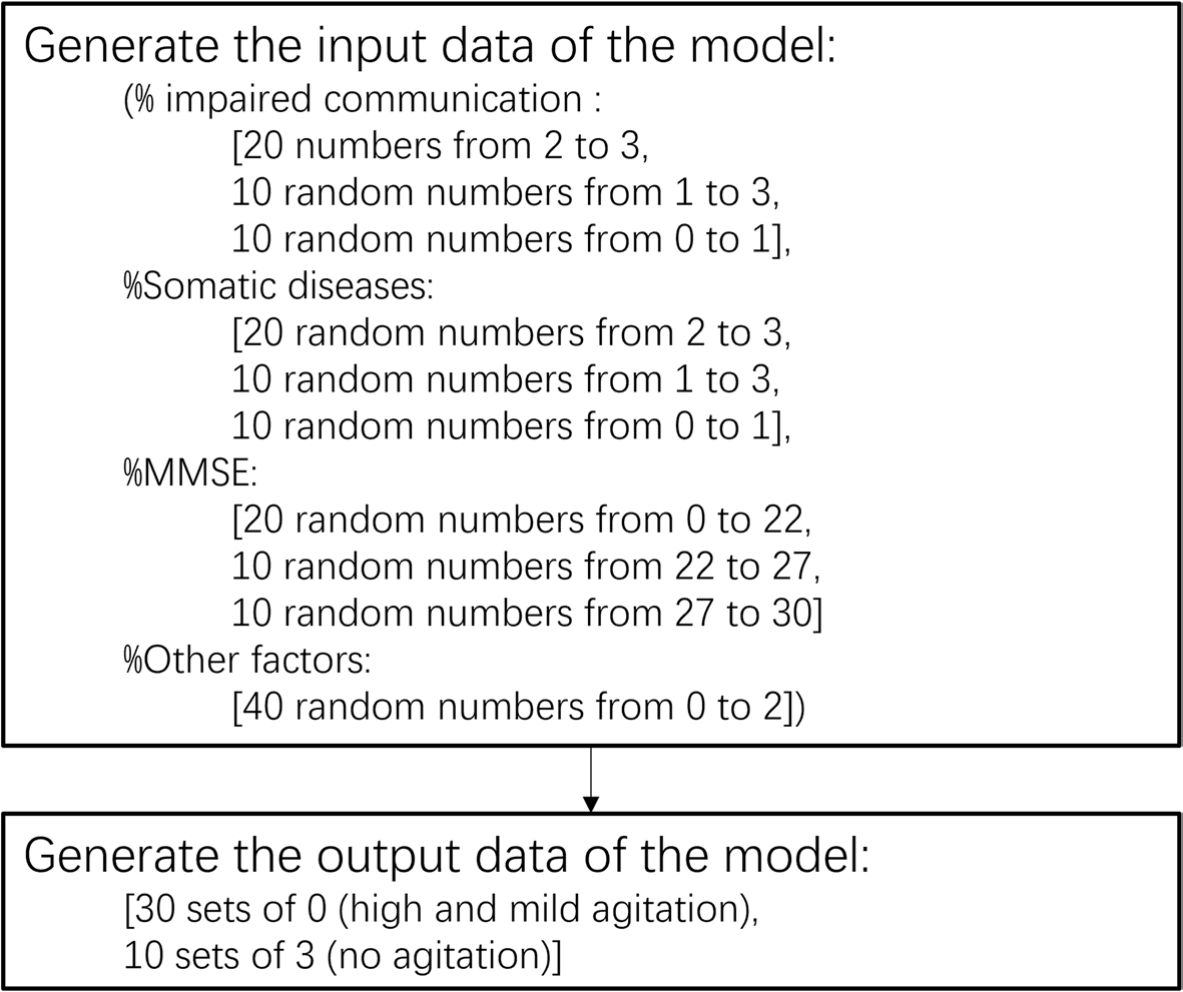
The pseudocode for generating data for training the PsyCo BPSD (agitation) model

Based on these regularities, 120 data samples, 40 for agitation, 40 for depression, and 40 for apathy, were generated using the random number function. For each type of BPSD, there are 20 high, 10 mild, and 10 no symptoms samples. Figure 2 shows the pseudocode for the generation of the agitation data samples. Those of the depression and apathy types are generated using the similar pseudocode.

#### 2.2.5 To acquire the model

After the data were generated, they were fed into the classification learner application in MATLAB. The decision tree algorithm was chosen. The parameters in the decision tree include the splitting criterion, which is the Gini diversity index, and the maximum number of splits, which is 20. The other parameters were optimized by the application.

### 2.3 Construction of the BePhyEn BPSD model

Since the approach is same as that for the construction of the PsyCo BPSD model, which is detailed in Sect. 2.2, only the aspects different are explained in this sub-section.

#### 2.3.1 To select the behaviors, physiological states, and other factors for the model

The influence of BPSD on daily-living behaviors and the other manifestations has been reported by various researchers [9, 41–48]. Two selection criteria were employed: those reflecting the relationship between a manifestation and at least one of the three selected BPSDs, and those can be observed or measured by sensors in home environment. The following six manifestations are selected accordingly:

- ‘Time’ (a period time in a day).
- ‘Environmental changes’ (which can be obtained by comparing the current environment with the previous one [49])
- ‘Walking speed’ (which can be obtained by the mobile robot following the elderly individual).
- ‘Heart rate variability’ (which can be obtained by millimeter wave radar [50, 51])
- ‘Daily activity’ (which can be reflected by walking distance or activity identification techniques [52, 53])
- ‘Wandering behavior’ (which can be obtained by checking trajectory of the elderly individual [54]).

The relationships between these manifestations and BPSD are summarized in Table 4.

**Table 4:** The relationship between manifestation and BPSD

Behavior and other manifestations	Agitation	Apathy	Depression
Time [41, 42]	○		○
Environmental change [43]	○		○
Walking speed [44–46]	○	○	○
Heart rate variability (HRV) [47]	○		○
Daytime activity [48]		○	
Wandering behavior [9]			○

○indicates that this factor affects this type of BPSD

HRV in Table 4 is an important physiological parameter. Physiological responses are controlled by the autonomic nervous system (ANS), which is subdivided into the sympathetic nervous system and the parasympathetic nervous system. Heart rate variability (HRV) is the fluctuation between successive heartbeat cycles. Studies in psychology and neuroscience have confirmed that cyclical variations in heart rate are caused by a continuous interaction between the sympathetic and parasympathetic nervous systems, which can be reflected by HRV measurements [47]. As shown in [55, 56], the parasympathetic activity is significantly reduced and HRV is low in depressive state. Whereas agitation was associated with higher sympathetic activity and exhibited higher HRV index values [57].

#### 2.3.2 To discretize the manifestations

Although the manifestation selected are naturally quantitatively, they need to be discretized so that these manifestations are under the same order of magnitude and also contribute to the understanding for the care givers or home care providers. The discretization of the manifestations is shown in Table 5.

**Table 5:** Discretization of manifestation

Manifestation	Discretization	Evidence
Time	xϵ (9,12) o’clock→’0’; xϵ (12,15) o’clock→’1’; xϵ (15,17) o’clock→’2’; xϵ (17,19) o’clock→’3’; xϵ (19,21) o’clock→’4’; others→’5’	[9, 41, 42, 58, 59]
Walking speed	xϵ (0,0.7) m/s→’0’; xϵ (0.7,0.9) m/s→’1’; xϵ (0.9,1.2) m/s→’2’; xϵ (1.2,1.5) m/s→’3’	[60–63]
Environmental change	[0 1 2 3] for [Nought Low Medium High]	[8]
Heart rate variability (HRV)	[0 1 2 3] for [Nought Low Medium High]	[8]
Daytime activity	[0 1 2 3] for [Nought Low Medium High]	[8]
Wandering	[0 1] for [Nought exist]	

x is the value of this manifestation

‘Time’: [42] showed that the temporal pattern of total agitation found showed a gradual increase from morning to approximately 4 p.m. and a gradual decrease thereafter. So 3 to 5 p.m. was set as the time period when aggression is more likely to occur (Time ‘2’), and 9 a.m. to 12 p.m. was set as the morning period (Time ‘0’) [58, 59]. showed a relationship between the sundowning effect and depression and [9] showed the correlation between depression and wandering behavior. Wandering behavior is often seen from 5 to 7 p.m [41]. . So 5 to7 p.m. was set as the time period when wandering may occur (Time ‘3’), and 5 to 9 p.m. was set as the sundowning period (Time ‘3–4’).

‘Walking speed’: studies have shown that the walking speed of normal elderly people is about 0.97 m/s [60], slower walking speeds indicate a higher probability of dementia [61, 62]. Some studies have classified walking speed into 4 intervals as < 0.6 m/s, 0.6 ~ 0.8 m/s, 0.8 ~ 1 m/s, > 1 m/s [63]. In this study, 4 intervals as < 0.7 m/s, 0.7 ~ 0.9 m/s, 0.9 ~ 1.2 m/s, > 1.2 m/s was set represent very lower, lower, normal, higher walking speed respectively.

‘Environmental change’, ‘HRV’, ‘Daytime activity’: The discretization is similar to Table 2.

#### 2.3.3 To determine the regularity of manifestation level range

The regularities between the discretized manifestations and the three levels of BPSD are shown in Table 6. The regularities and the evidence for them are described as follows:

- ‘Time’: Since the agitation tends to occur in the afternoon during the day and peaks at 4 p.m [42]. , the time period of high agitation was set to 3 to 5 pm, i.e., Time ‘2’. The mild agitation time was set to 9 am to 5 pm, i.e., Time ‘0–2’. It had been shown that the wandering behavior mainly occurs from 5 to 7 p.m [41]. Therefore, the high depression patient will have wandering behavior in Time ‘3’. Mild depression will more likely occur from 3 to 9 p.m., i.e., Time ‘2–4’.
- ‘Environmental changes’: Environmental changes can exacerbate agitation and depression [43]. Same as the Sect. 2.2.3, the drastic environmental changes are connected to severe agitation and depression.
- ‘Walking speed’: Slower walking speeds indicate a higher probability of dementia [62]. According to this fact, the high BPSD, mild BPSD and no BPSD would have speed ‘0’, ‘0–2’, and ‘2–3’ respectively.
- ‘HRV’: It has been shown that depression was negatively correlated with HRV [55, 56]. Thus, the HRV for high depression was set to ‘0–1’, mild depression to ‘0–2’ and no depression to ‘2’. Whereas HRV was higher in agitation [57], thus, for high aggression, mild agitation and no aggression, the HRV was set to ‘3’, ‘2–3’, ‘2’, respectively.
- ‘Daytime activity’: Cognitive impairment reduces daytime activity, and apathy evaluation scale (AES) scores has significantly correlation with daytime activity [48]. Therefore, high apathy, mild apathy and no apathy, the Daytime activity was set to ‘0–2’, ‘1–2’, ‘2–3’, respectively.
- ‘Wandering’: Wandering has correlation with depression [9]. Therefore, high depression and mild depression were set to wandering ‘0–1’.

#### 2.3.4 To generate the data for training the BePhyEn BPSD model

**Fig. 3:**
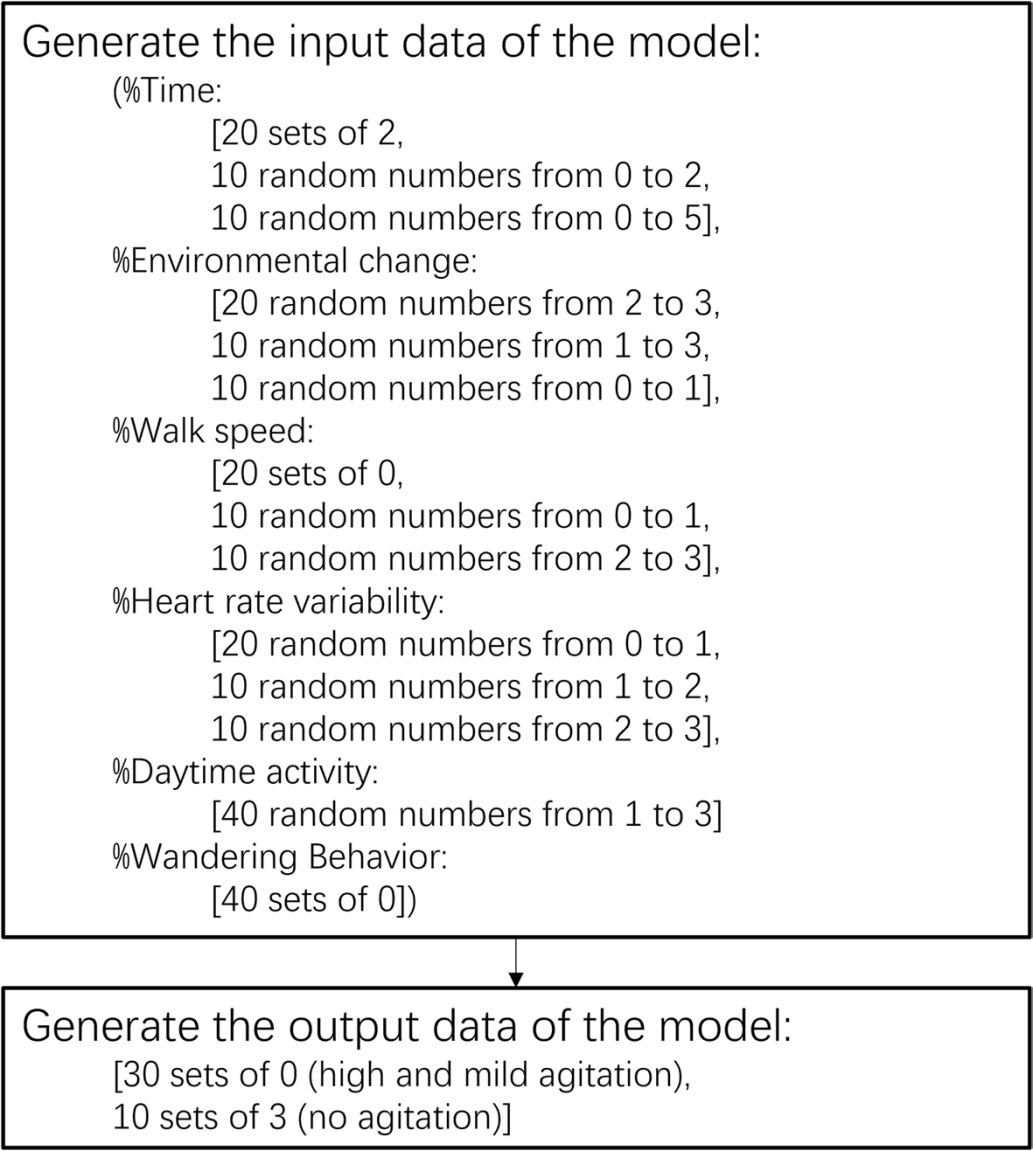
The pseudocode for generating data for training the BePhyEn BPSD (agitation) model

As described in Sect. 2.2.4, 120 sets of data with the same composition were generated. Figure 3. shows pseudocode for generating the data of the agitation type, and the other two BPSD data were generated using similar procedures.

#### 2.3.5 To compare the PsyCo BPSD model and BePhyEn BPSD model with and without HRV factor

HRV is usually represented by changes in the RR interval from the electrocardiogram (ECG). Commonly used HRV indices include normalized low frequency power (LFn), normalized high frequency power (HFn), and standard deviation of normal-normal intervals (SDNN) [47]. Studies have shown that during happy states, the average heart rate, SDNN, and LFn are higher than during sad states, while HFn is the opposite, indicating that sympathetic nervous system activity is greater and parasympathetic nervous system activity is smaller during happy states [64]. In the field of dementia research, a study used HRV to assess influence to the sympathetic nervous system of dementia patients, showing statistically significant reductions in HFn in the disease group in both supine and standing positions [65].

Thus, it is expected that, HRV could be an important factor for the BPSD identification. Moreover, it is necessary to discuss whether the same effect can be achieved by behavior identification without HRV in BePhyEn BPSD model, and whether it can lead to improvement in PsyCo BPSD model. Therefore, a BePhyEn BPSD model without using HRV and a PsyCo BPSD model with HRV were built to further investigate the significance of HRV.

### 2.4 Elderly individual following algorithms

**Fig. 4:**
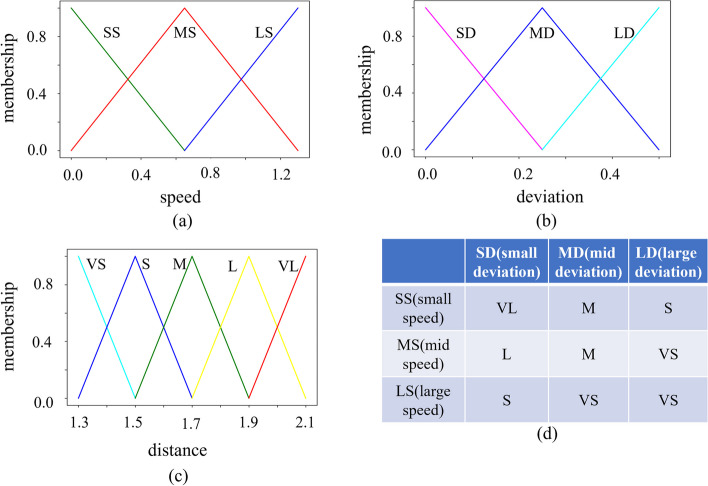
Fuzzy membership functions and fuzzy rules. **a** membership chart of speed, ranging from 0 to 1.3. SS: small speed, MS: middle speed, LS: large speed. **b** membership chart of deviations, ranging from 0 to 0.5. SD: small deviation, MD: middle deviation, LD: large deviation (**c**) membership chart of distance output, for which different output values were set depending on the room. VS: very small distance, S: small distance, M: middle distance, L: large distance, VL: very large distance (**d**) control rules. When the target speed is faster, the closer the robot follows, and vice versa, the farther it is. When the deviation of the target is larger, the closer the robot follows, and vice versa

Three following algorithms were selected for comparison. Algorithm 1 is a built-in following program, in which the robot constantly follows the target. However, if the target only moves within a small range, the robot needs to move all the time, which leads to more power consumption or collision risks. This is why Algorithm 2 was proposed. However, Algorithm 2 may lose the target due to sudden turning and long following distance. Therefore, an algorithm that can automatically adjust the following distance is needed. It has been reported that fuzzy logic control algorithm can enable decisions making in complex environments with a simple and light model and at the same time can perform effective path planning [66, 67]. The details of the algorithms are shown as follows:

- **Algorithm 1**: The robot follows the target at a certain distance from the target. If the target cannot be detected, it just stops until the target is detected.
- **Algorithm 2**: The robot keeps a range of distance from the target. The robot adjusts the distance to the target in real time to ensure that the distance can be within the set range. Aso, if the target cannot be detected, it just stops until the target is detected.
- **Algorithm 3**: If the target walks faster or if the deviation of target from robot vision center is bigger, the robot is more likely to lose the target. In this situation, a closer following distance can effectively reduce the probability of losing. The fuzzy logic control algorithm implemented was adapted from that in [68] and the parameters are set as follows:

- Two input variables were defined: the speed of the target, deviation of target from robot vision center.
- One output variable was defined: the distance between the robot and the target during following.
- The fuzzy membership functions and rules were shown in Fig. 4.

## 3 Experiment settings

**Fig. 5:**
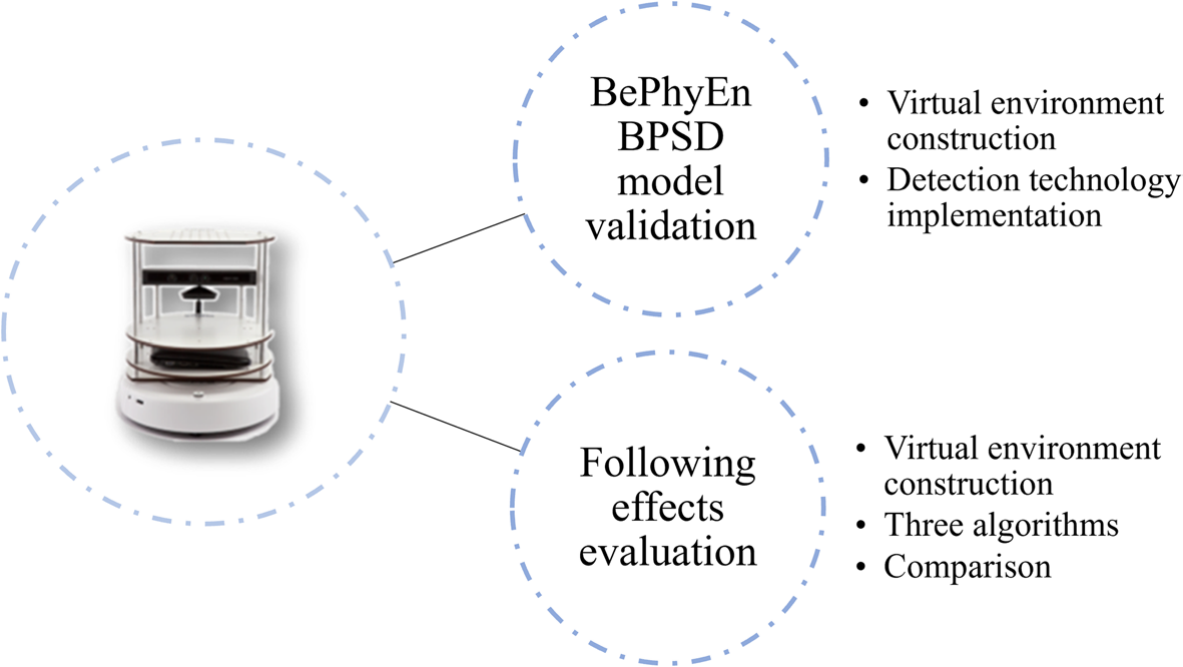
Robot monitoring platform

After establishing the two models, the experiments using the robot monitoring platform combined with the BePhyEn BPSD model in virtual home environments were conducted, as shown in Fig. 5.

To validate the BePhyEn BPSD model. Firstly, the Unity engine was used to build a virtual home environment. In the virtual home environment, a target (elderly individual) living alone, and a monitoring robot were designed. The detail of the virtual home environment construction was shown in Supplement 1–2. Then the data of the BPSD which was used for testing model was simulated in the virtual home environment. In the experiment, 120 data examples (30 agitation data samples, 30 apathy data samples, 30 depression data samples, and 30 no BPSD data samples) were generated using the regularity determined in Table 6. Finally, the BePhyEn BPSD model proposed was implemented and used by the robot to identify which BPSD type the target is at each moment.

**Table 6:** Regularities between the discretized manifestations and the three levels of BPSD. The items in yellow, orange, blue represent agitation, apathy, and depression-related manifestations, respectively

	High agitation	Mild agitation	No agitation	High apathy	Mild apathy	No apathy	High depression	Mild depression	No depression
Time	2	0–2	0–5	0–5	0–5	0–5	3	2–4	0–5
Environmental change	2–3	1–3	0–2	0–2	0–2	0–2	2–3	1–3	0–2
Walking speed	0	0–2	2–3	0	0–2	2–3	0	0–2	2–3
Heart rate variability(HRV)	3	2–3	2	2	2	2	0–1	0–2	2
Daytime activity	1–3	1–3	1–3	0–2	1–2	1–3	1–3	1–3	1–3
Wandering Behavior	0	0	0	0	0	0	0–1	0–1	0

To investigate the effectiveness of the robot following in different home environments, first, three rooms were constructed, room 1 with a larger area and fewer occlusions, room 2 with a smaller area and more occlusions and room 3 which was based on room 1 with more occlusions, which was shown in Supplement 2. A fixed roadmap was set up in each room, ensuring that there are no variables other than the following algorithms. Power consumption rate, Mean Absolute Error (MAE), lost time, and collision time were used as performance indexes to compare the performance of the three following algorithms.

### 3.1 BePhyEn BPSD model validation

There are many sensing and measurement modules that can be loaded on a mobile robot. In this study, considering practical meaning of verification, only some of them were implemented for the virtual experiments, and the others, such as, time, environmental change, HRV, daytime activity were assumed to be known. The detection of walking speed and wandering behavior were implemented as follows:

- **Speed detection**.

Speed of the target, in the unity, was calculated by recording the change in the target’s position. In Unity, the position and angle of the robot can be obtained. The robot position in time $t$ was set as $\left(XR\right(t), YR(t\left)\right)$ and the angle of the robot in time $t$ was set as$angle\left(t\right)$. The distance between the robot and the target in time $t$ was set as $distance \left(t\right)$. The position of the target in time $t$ was set as $\left(XT\right(t), YT(t\left)\right)$. The following formula can be used to calculate the target’s position at time *t*.

1 $$XT\left(t\right)=XR\left(t\right)+distance\left(t\right)\text{*cos}\left(angle\right(t\left)\right)$$

2 $$YT\left(t\right)=YR\left(t\right)+distance\left(t\right)\text{*sin}\left(angle\right(t\left)\right)$$

3 $$V\left(t\right)=\sqrt[2]{{(XT\left(t+1\right)-XT(t\left)\right)}^{2}+{(YT\left(t+1\right)-YT(t\left)\right)}^{2}}$$

The speed of the target *V(t)* is determined by calculating its movement per second. As there is up-and-down in the speed of the robot during the target following, the average value of every 5 s was used for judgement.

- **Wandering detection**.

**Fig. 6:**
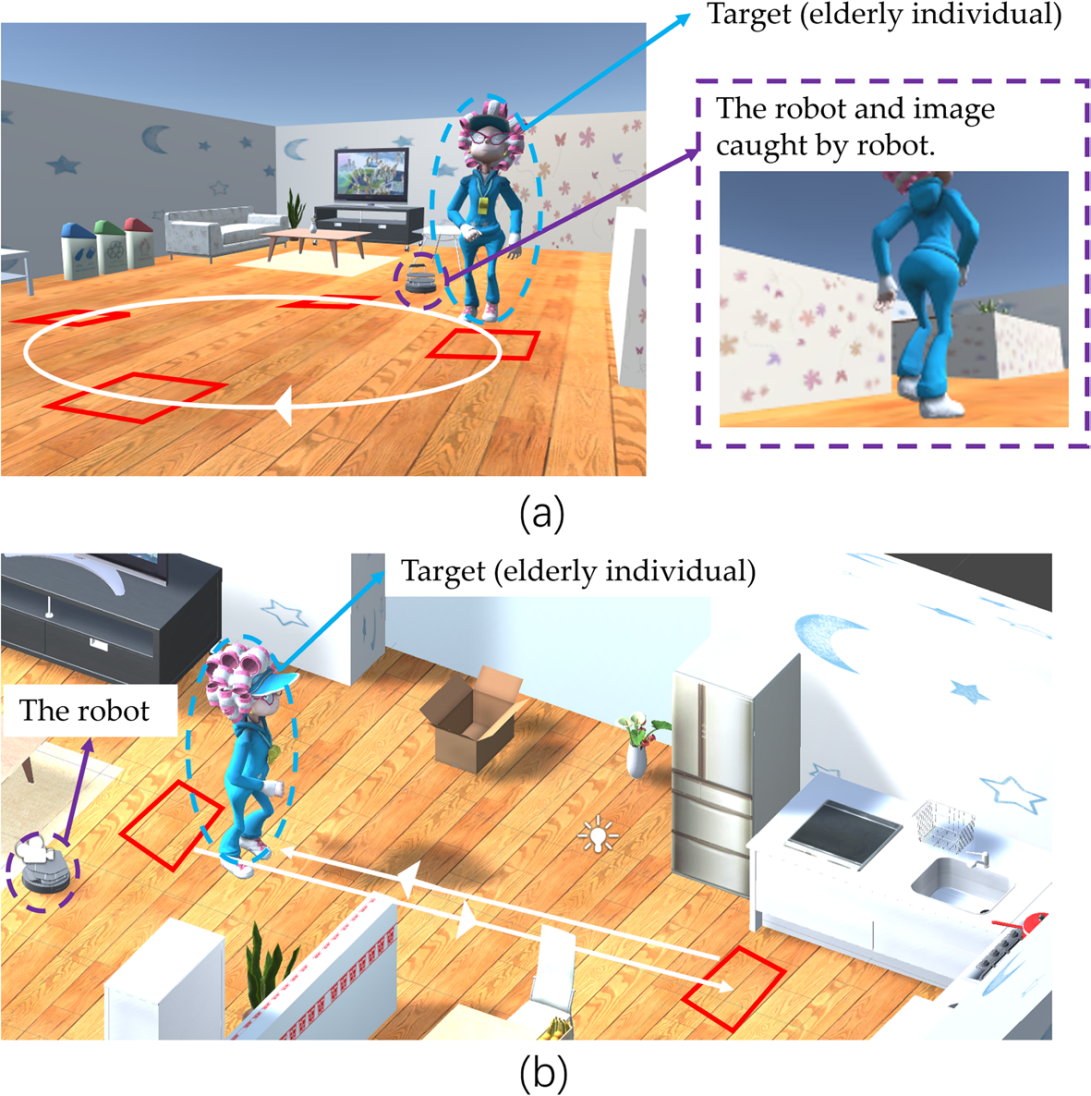
Wandering behavior simulation. Red rectangle: mark position. Blue: the target (elderly individual); Purple: the robot and image caught by robot. (**a**) a white circle: a typical lapping route. (**b**) white lines: a typical pacing route

There are many wandering behaviors including lapping, pacing and random [69]. In this study, the lapping and pacing were simulated for the virtual experiments. A typical lapping route was shown with a white circle in Fig. 6.(a). A typical pacing route was shown with white lines in Fig. 6.(b). To detect this wandering behavior in the home environment, rectangles were set for counting the times of arrival. As shown in Fig. 6.(a). When the target traverses a rectangle, the rectangle is marked, and when the target traverses four rectangles in sequence, the target is considered to have made a circle once. When the target circled four times, it was considered wandering behavior. In Fig. 6.(b), there are two rectangles.

### 3.2 Target following algorithm evaluation

For comparison and analysis of the algorithms, some experimental procedures were set to evaluate the performance of each algorithm in different rooms.

#### 3.2.1 To set the scenarios:

A process was designed as the target goes back home to eat in the dining room, then goes to the living room to watch TV, and finally goes to the bedroom for rest. The target walked at different speeds of 0.6, 0.67, 0.74, 0.81, 0.87, 0.94, 1.01, 1.08, 1.14, and 1.2, respectively. Each algorithm tests ten walking speeds in each room.

#### 3.2.2 To determine the maximum and minimum following distance:

The maximum and minimum distance were determined for the robot to follow. It has been found that the personal following distance between the robot and target is 45–120 cm, and the social following distance between the robot and target is 120–360 cm. Usually, people prefer the social follow distance, and those with a high affinity with robots prefer a closer distance [70]. In this study, it was considered that the robot needed to achieve a better following effect within the following range and would not easily lose the target. So, for different rooms, different following distances were set as follows:

- Room1: maximum distance: 2.1 m, minimum distance: 1.3 m.
- Room2: maximum distance: 1.8 m, minimum distance: 1.3 m.
- Room3: maximum distance: 2.1 m, minimum distance: 1.3 m.

#### 3.2.3 Four performance indexes:

Finally, four indexes were set to compare the following algorithms:

4 $$Power\ consumption\ rate =\frac{robot\ distance\ travelled}{human\ distance\ travelled}$$

5 $$MAE = \frac{1}{m}{\sum }_{i=1}^{m}\left|{y}_{i}-\widehat{{y}_{i}}\right|$$

- Lost times: the number of times the target is lost in ten tests.
- Collision times: the number of times the robot collided with a person or a wall among the ten tests.

Power consumption rate is the ratio of walking distance between robot and human. The smaller the value means the less power consumption for following. And the MAE is used to calculate the speed detection accuracy. The lost time is the number of times the robot lost the target during ten tests. The collision time is the number of collisions that occurred during ten tests.

In the whole experiment, the target would walk according to the fixed roadmap, and three following algorithms were applied to make the robot follow the target, and then the four indexes were calculated to analyze the following performance.

## 4 Results

### 4.1 The results of three following algorithms

Each following algorithm was tested ten times in each room, and the average of power consumption rate and MAE were calculated. The lost times and the collision times were summed. The comparison results of the three algorithms are shown in Tables 7, 8 and 9.

**Table 7:** Comparison regarding room 1

Room 1	Algorithm 1	Algorithm 2	Algorithm 3
Power consumption rate	0.75	0.69	0.69
MAE	0.11	0.14	0.11
Lost times	0	2	0
Collision times	0	1	0

**Table 8:** Comparison regarding room 2

Room 2	Algorithm 1	Algorithm 2	Algorithm 3
Power consumption rate	0.67	0.62	0.63
MAE	0.12	0.14	0.13
Lost times	1	1	1
Collision times	0	0	0

**Table 9:** Comparison regarding room 3

Room 3	Algorithm 1	Algorithm 2	Algorithm 3
Power consumption rate	0.8	0.78	0.78
MAE	0.19	0.18	0.18
Lost times	3	3	2
Collision times	1	1	1

In the room 1, as shown in Table 7, Algorithm 1 has a better MAE value, and results in no collisions and no cases of lost-target. But the robot has a higher power consumption rate. On the other hand, Algorithm 2 is more power efficient than Algorithm 1. But there are more cases of lost-target. Algorithm 3 shows the performance no worse than the other two algorithms.

Table 8 shows the comparison results in room 2. As shown, in a narrow room, because there were more occlusions (walls, furniture), and the target has to turn more frequently, the robot with all the three algorithms is more likely to lose the target when the target moves with a high speed. However, the MAE value did not change greatly, which means the speed detection accuracy was still satisfactory. In terms of power consumption rate, Algorithms 2 and 3 have advantages over Algorithm 1.

Table 9 shows the comparison regarding room 3. As shown in the table, the room is large, but with more occlusions (furniture), so the robots of all three algorithms are likely to lose the target when the target is turned at high speed. In addition, the velocity detection accuracy in room 3 is lower than that of room 1.

### 4.2 The two PsyCo BPSD model and two BePhyEn BPSD models

**Fig. 7:**
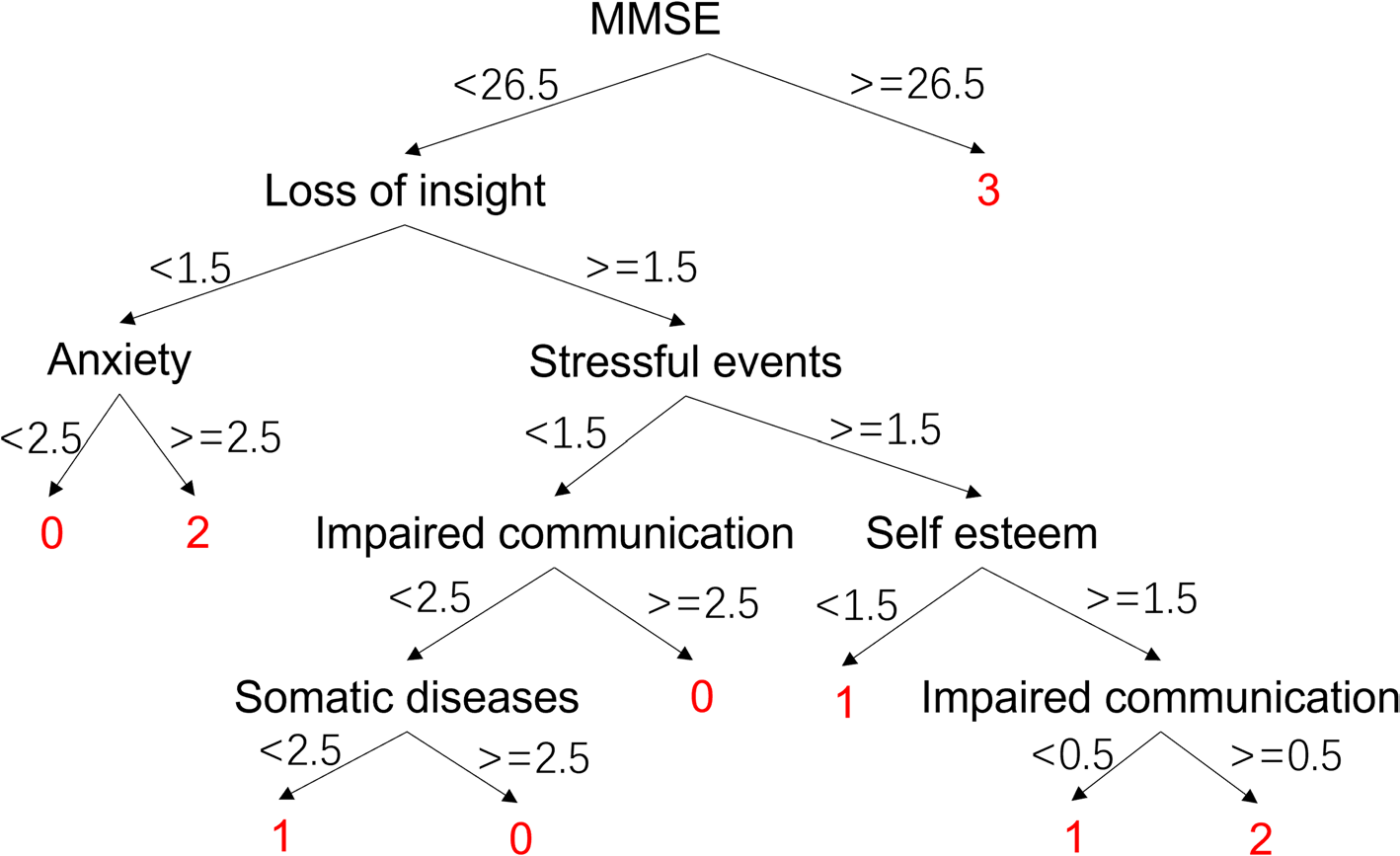
PsyCo BPSD model. 0: Agitation, 1: Apathy, 2: Depression, 3: no BPSD. Please see Sect. 2.2.2 for the meaning of the factors

The final decision tree model obtained was shown in Fig. 7, which can be used to identify BPSD using assessment of psychological and social characteristics by care professionals and care staff. MMSE has the largest contribution in the model. When MMSE > 26.5, the result of the model discrimination was no BPSD symptoms. And if MMSE < 26.5, agitation and depression behaviors can be judged by loss of insight and anxiety.

**Fig. 8:**
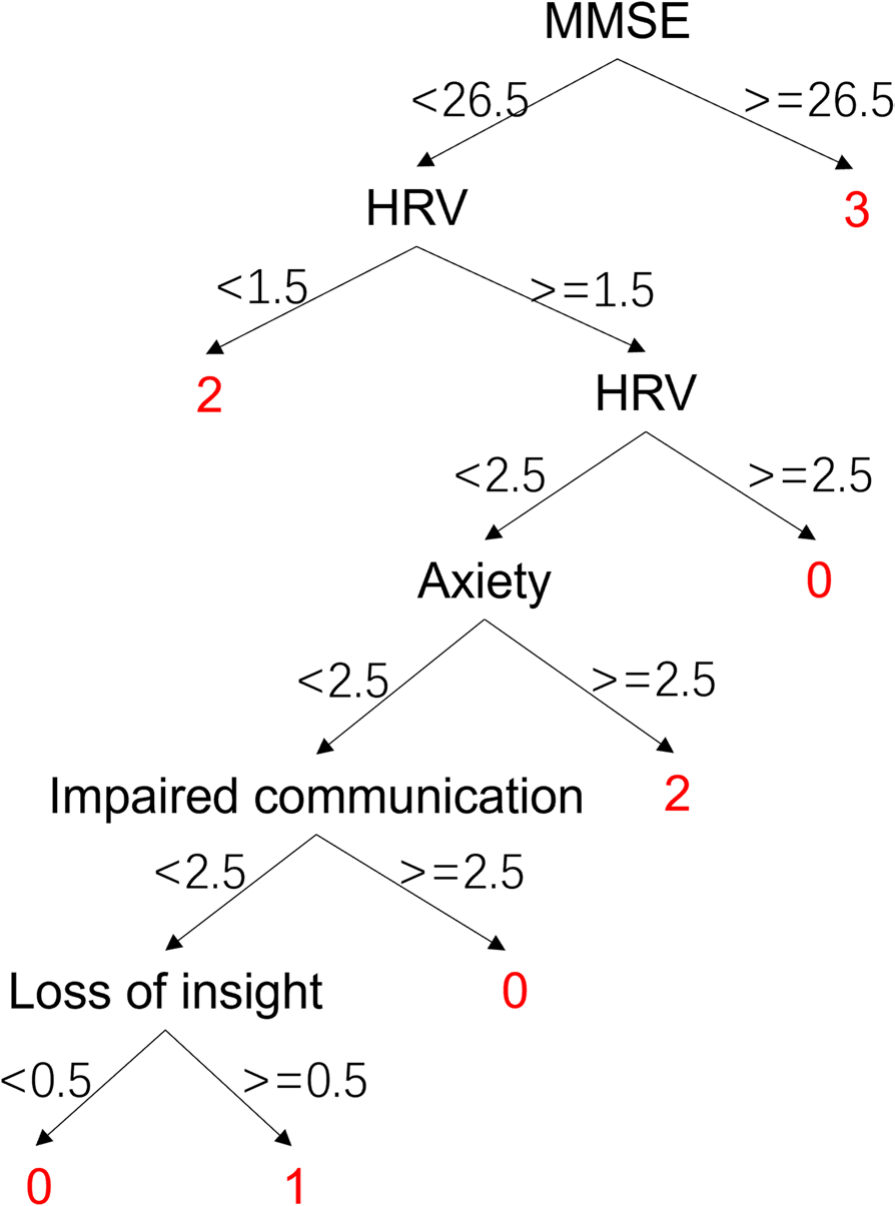
PsyCo BPSD model with manifestation HRV. 0: Agitation, 1: Apathy, 2: Depression, 3: no BPSD. Please see Sect. 2.2.2 for the meaning of the factors

PsyCo BPSD model with the manifestation HRV is depicted in Fig. 8. Same as the PsyCo BPSD model, MMSE shows the highest importance, and if MMSE is greater than 26.5 then no BPSD is determined. If MMSE is less than 26.5 then HRV, ranked the second in importance, is the next attribute to judge. If HRV is lower than 1.5, the model gives judgement of depression.

**Fig. 9:**
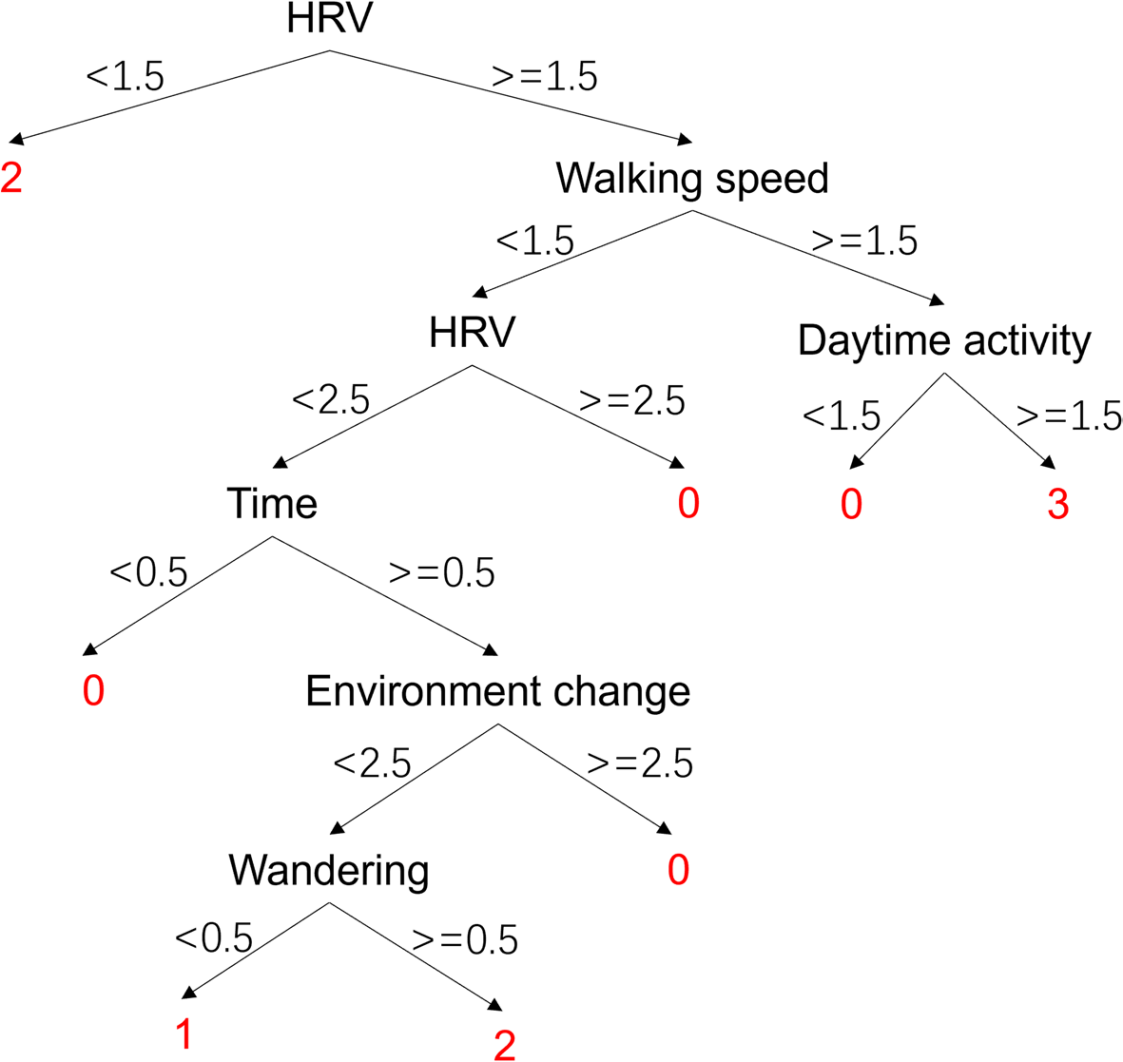
BePhyEn BPSD model. 0: Agitation, 1: Apathy, 2: Depression, 3: no BPSD. Please see Sect. 2.3.2 for the meaning of the attributions

The BePhyEn BPSD model is shown in Fig. 9. In this model, the importance of HRV manifestation is the highest, which is in line with the previous analysis. A lower HRV indicates a lower parasympathetic activity thus the depression can be identified. Walking speed was the second important attribute. The higher walking speed and higher daytime activity are likely to indicate no BPSD.

**Fig. 10:**
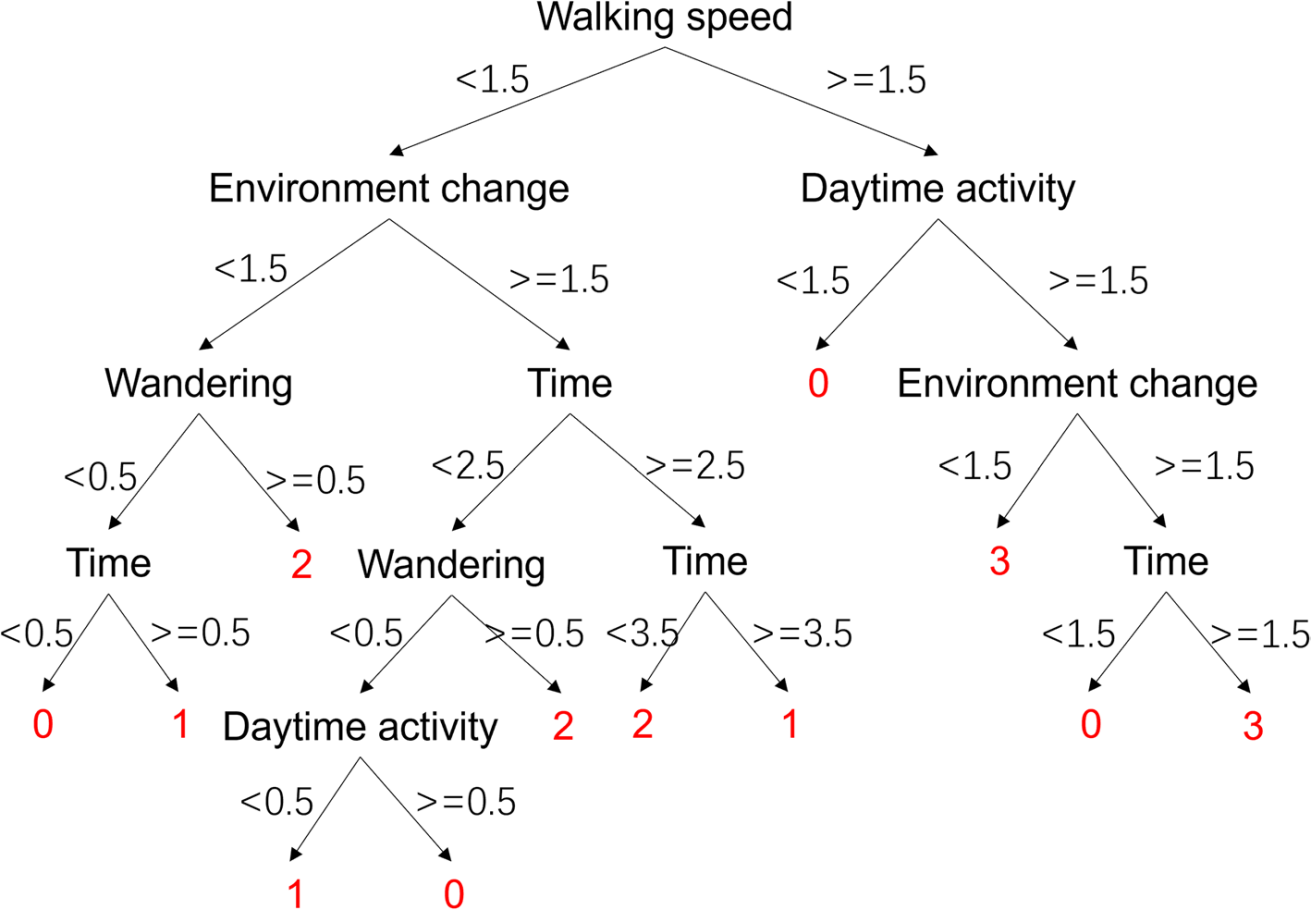
BePhyEn BPSD model without manifestation HRV. 0: Agitation, 1: Apathy, 2: Depression, 3: no BPSD. Please see Sect. 2.3.2 for the meaning of the attributions

The BePhyEn BPSD model without manifestation HRV is shown in Fig. 10. Different from the BePhyEn BPSD model, walking speed became the first important manifestation. Both the BePhyEn BPSD model and this model give the judgment of no BPSD only when the walking speed is normal. This reflects that slow walking speed is an important index of dementia.

### 4.3 The validation of the models using the data generated following BPSD regularities

Table 10 presents the validation results using 120 data samples generated for testing the four models. For each model, its testing data samples were generated in the same way with the same composition as the training data samples. The validation accuracy of the models was all above 80%. In particular, the validation accuracy of the PsyCo BPSD model with manifestation HRV can reach more than 90%.

**Table 10:** Model validation result

Model	Validation Accuracy
PsyCo BPSD model	82.5%
PsyCo BPSD model with manifestation HRV	93.3%
BePhyEn BPSD model	89.2%
BePhyEn BPSD model without manifestation HRV	85%

Table 11 shows validation results of the BePhyEn BPSD model for 25 samples (all (13) incorrect cases and part of (12, 3 for each BPSD label) correct cases).

**Table 11:** validation results of 25 data samples. Red: the incorrect cases. Pink: further discussed in Sect. 5.1. Please see Sect. 2.3.2 for the meaning of the attributions

Number	Walking speed	Environment change	Wandering	Time	Daytime activity	HRV	BPSD labels	Validation results
1	1	1	0	0	2	2	0	0
2	1	2	0	0	2	2	0	0
3	2	2	0	1	1	2	0	0
4	0	1	0	1	2	2	1	1
5	1	2	0	4	2	2	1	1
6	1	2	0	4	2	2	1	1
7	1	3	0	4	2	1	2	2
8	0	3	1	3	1	1	2	2
9	0	2	1	3	2	2	2	2
10	2	2	0	2	2	2	3	3
11	2	1	0	5	2	2	3	3
12	3	2	0	2	3	2	3	3
13	1	2	0	2	2	2	0	1
14	2	2	0	1	2	2	0	3
15	2	2	0	0	2	2	0	3
16	2	2	0	0	1	2	1	0
17	2	2	0	1	2	2	0	3
18	2	2	0	0	1	2	1	0
19	0	2	0	3	2	2	2	1
20	2	1	0	2	2	2	1	3
21	0	1	0	0	2	2	1	0
22	2	1	0	3	1	2	1	0
23	0	2	0	0	0	2	1	0
24	1	3	0	3	2	2	2	0
25	2	3	1	2	1	2	2	0

### 4.4 The validation of BePhyEn BPSD model using the data acquired in virtual home environment

In the virtual home environment experiment, the Algorithm 3 was chosen for testing the BePhyEn BPSD model. In room 1, the lapping route of wandering behavior was tested. In room 3, the pacing route of wandering behavior was tested. Table 12 shows the validation results of 24 (6 for each BPSD label) of the 120 data samples for the robot to identify, ground truth BPSD labels, and the identification results by the BePhyEn BPSD model. The results indicate that target following and the speed detection in room 1 was effective. However, in room 2 and room 3, the robot tends to lose the target due to more occlusions, especially when the target is walking faster than 1.2 m/s. Generally, the BePhyEn BPSD model achieved identification accuracy of 80% for the 120 data samples in the virtual home environment experiment.

**Table 12:** validation results of 16 data samples acquired in the virtual home environment. Red: incorrect cases. Light green: correct recognition cases in room (1) Green: correct recognition cases in room (2) Deep green: correct recognition cases in room (3) Please see Sect. 2.3.2 for the meaning of the attributions

Virtual home environment	Walking speed	Detected walking speed	Environment change	Wandering	Detected wandering	Time	Daytime activity	HRV	BPSD labels	Identify results
Room 1	1 (0.8 m/s)	1 (0.76 ~ 0.87 m/s)	3	0	0	2	2	2	0	0
	0 (0.5 m/s)	0 (0.45 ~ 0.55 m/s)	3	0	0	2	2	3	0	0
	0 (0.5 m/s)	0 (0.45 ~ 0.55 m/s)	0	0	0	1	0	2	1	1
	0 (0.6 m/s)	0 (0.57 ~ 0.63 m/s)	2	0	0	0	0	2	1	0
	2 (1.0 m/s)	2 (0.99 ~ 1.04 m/s)	2	1	1	3	1	0	2	2
	1 (0.8 m/s)	1 (0.76 ~ 0.87 m/s)	1	1	1	2	2	0	2	2
	3 (1.25 m/s)	3 (1.18-1.35 m/s)	0	0	0	2	2	2	3	3
	2 (1.1 m/s)	2 (1.04 ~ 1.18 m/s)	1	0	0	0	3	2	3	3
Room 2	0 (0.5 m/s)	0 (0.45 ~ 0.57 m/s)	3	0	0	2	1	3	0	0
	0 (0.6 m/s)	0 (0.55 ~ 0.66 m/s)	2	0	0	1	2	3	0	0
	1 (0.8 m/s)	1 (0.73 ~ 0.89 m/s)	2	0	0	4	2	2	1	1
	2 (1.0 m/s)	2 (0.90 ~ 1.05 m/s)	1	0	0	2	2	2	1	3
	0 (0.5 m/s)	0 (0.45 ~ 0.57 m/s)	2	0	0	3	3	1	2	2
	1 (0.8 m/s)	1 (0.73 ~ 0.89 m/s)	3	0	0	3	2	2	2	0
	2 (1.1 m/s)	2 (0.98 ~ 1.07 m/s)	1	0	0	0	3	2	3	3
	3 (1.2 m/s)	lost	2	0	0	2	3	2	3	lost
Room 3	0 (0.5 m/s)	0 (0.45 ~ 0.55 m/s)	2	0	0	1	3	3	0	0
	2 (1.0 m/s)	1 (0.97 ~ 1.12 m/s)	3	0	0	2	2	2	0	3
	0 (0.55 m/s)	0 (0.53 ~ 0.58 m/s)	1	0	0	2	2	2	1	1
	2 (1.1 m/s)	1 (0.97 ~ 1.2 m/s)	2	0	0	4	1	2	1	0
	0 (0.5 m/s)	0 (0.46 ~ 0.58 m/s)	3	1	1	3	1	0	2	2
	2 (1 m/s)	lost	1	1	0	2	1	2	2	lost
	2 (1 m/s)	2 (0.97 ~ 1.06 m/s)	2	0	0	2	3	2	3	3
	3 (1.2 m/s)	lost	1	0	0	3	2	2	3	lost

## 5 Discussion

In this study, a BPSD monitoring system is presented, which consists of two models and a monitoring platform. The PsyCo BPSD model enables caregivers and care providers to make a preliminary judgement of BPSD through observation. On the other hand, the BePhyEn BPSD model can be implemented on a robot to achieve real-time warning of BPSD. It was implemented and validated on a robot in virtual home environment. Additionally, three following algorithms were also compared for better following performance in the virtual home environment.

### 5.1 BPSD model

In PsyCo BPSD model, the second importance was given to loss of insight, which denotes the psychological symptom that an elderly individual is unable to recognize changes in behaviour and personality. Loss of insight was used as a diagnostic criterion for frontotemporal dementia (FTD) [71]. While in our model, loss of insight is an important indicator to distinguish the agitation. In the BePhyEn BPSD model, HRV was found to be a significant factor. The identification accuracy is higher than that of the model without HRV by approximately 4.2%, which aligns with the physiological interpretation. HRV is generally obtained by measuring beat-to-beat heartbeat interval. We have made progress in applying millimeter-wave radar for mobile sensing to detect heartbeats [51], which made the non-constraint monitoring of HRV feasible in home environment.

The validation results showed that the accuracy of PsyCo BPSD model was lower than that of BePhyEn BPSD model (Table 10). This is due to the following reasons: (1) Parameter selection: MMSE, the primary parameter of the PsyCo BPSD model (Fig. 7), intuitively represents the mental state of an older person, but doesn’t contain much information about BPSD. Whereas HRV, the primary parameter of the BePhyEn BPSD model (Fig. 9), contains significant information for identifying BPSD types such as aggression or depression. When the manifestation HRV was added to PsyCo BPSD model, the accuracy of the model reached 93.3% (Table 10); (2) The regularities of data generation: in this study, the data for training and validation were generated by regularities determined from numerous relevant literatures. In the literature related to PsyCo BPSD model, most of findings are qualitative. Therefore, more probabilistic ambiguity needs to be accounted in the regularities, which is more likely to cause overlapping value range of factors across different BPSDs. In contrast, in BePhyEn BPSD model related literature, manifestations such as time, walking speed, HRV, etc. are quantitatively and clearly defined, which resulted in clear regularities, thereby, better data samples for training and validating the models.

Regarding the BePhyEn BPSD model (as shown in Fig. 9), all the incorrect identification cases were shown for discussion (Table 11). The first type of incorrect identification is that the data samples of different BPSD labels were judged as a same label because they have similar attribute values for the BePhyEn BSPD model to make the same judgement. For example, between No.5 and No.13, only Time attribute is different (4 vs. 2). The nodes and leaves these two samples undergone are as follows:

- No.5 (label 1): HRV ‘2’, Walking speed ‘1’, HRV ‘2’, Time ‘4’, Environment change ‘2’, Wandering ‘0’ ⇒ label ‘1’;
- No.13 (label 0): HRV ‘2’, Walking speed ‘1’, HRV ‘2’, Time ‘2’, Environment change ‘2’, Wandering ‘0’ ⇒ label ‘1’;

The second type of incorrect identification is that the data samples of different BPSD labels underwent different nodes and leaves of the BePhyEn BPSD model and ended up with a same label. For example, No.3 and No.24, have the different attributes in most manifestation attributes. The nodes and leaves they underwent are as follows:

- No.3: HRV ‘2’, Walking speed ‘2’, Daytime activity ‘1’ ⇒ label ‘0’;
- No.24: HRV ‘2’, Walking speed ‘1’, HRV ‘2’, Time ‘3’, Environment change ‘3’ ⇒ label ‘0’;

It should be noted that some of the first type of incorrect identification cases (samples colored pink in Table 11) were caused by the overlapping value ranged for the manifestation determined in regularities (Table 6). For example, in manifestation values in No.13, which takes values in the range of agitation (e.g., manifestation values in No.2) but also take values in the range of apathy (e.g., manifestation values in No.5). The reasons for this situation were considered as follows:

1. The overlapping of range of manifestation values of different BPSDs is large. Although the regularities for each BPSD (agitation, apathy, depression) related manifestations were determined (as shown in Table 6) based on references in the literature, for unrelated manifestations, a wide range was set with the expectation that the obtained model would exhibit strong robustness in diverse situations.
2. Insufficient number of data samples resulted less diversity in combinations of manifestation values, leading to the case that single leaf of decision trees contains multiple BPSD labels. A further analysis of 60, 120, 240, and 480 training data samples were performed. As a result, the training accuracy was 86.7%, 88.3%, 89.5%, and 91.2%, and the area under curve (AUC) were 0.87, 0.95, 0.96, and 0.96, respectively. Considering that more samples can achieve higher accuracy but also lead to complexity and overfitting of the decision tree, 120 samples were considered to be sufficient in this study.
3. An elderly individual has only one type of BPSD but has the manifestations in range of two BPSD. The BePhyEn BPSD model can only identify the BPSD from the observed manifestations, and more precise determination still needs further confirmation.

### 5.2 Virtual home environment experiment

The simulation of dementia behavior in a virtual home environment has advantages, as targets can display a variety of daily behavioral actions [60], and the parameters of these actions can be precisely controlled. In addition, it is easier to control environmental variables in the virtual home environment and eliminate unnecessary noise interference. Because the target following and manifestation detection (walking speed, wandering detection, colored in green in Table 12) in the virtual experiment achieved the accuracy sufficient for the BePhyEn model to identify most of the test cases, no further misidentification was caused by the mobile robot monitoring platform. Besides, considering that the limited number of variables to be tested, and number of training data samples, which is 120, it is sufficient to input 120 data samples (40 per room) for the virtual robot to test performance.

Two reasons were considered for the errors caused in virtual home environment experiments, the first was the same as the analysis of Table 11, and the other was due to robot following errors (lost-targets). In terms of following algorithms, the advantage of algorithm 3 is that it can automatically adjust the following distance according to the target’s walking speed and the deviation of target from robot vision center. Therefore, the robot doesn’t follow the target all the time, but with a reduced loss time and power consumption rate, as shown in Tables 7 and 8. However, algorithm 3 tends to lose the target when there is more occlusion and the target is turning fast, as shown in Tables 8, 9 and 12. For wandering detection, pacing route is more difficult to detect than lapping route. Because when detecting the lapping route, the robot only needs to follow behind the target. In pacing route, the robot was located in front of or to the side of the target, which requires a higher adaptation of the robot’s position, As shown in Table 12, When the target walking speed is 1 m/s, the lapping route (in room 1) can be detected, while the pacing route (in room 2) cannot be detected.

### 5.3 Contribution

1) For the first time, multi-variate hierarchical models for identify three BPSDs (Agitation, Depression, Apathy) were proposed and verified. The models are represented as decision trees, trained with data sets generated based on a systematic analysis of the amount of data about BPSD manifestations in the literature.

2) Through validation of the BePhyEn BPSD model, it is clearly shown that, internal psychological states of elderly individual can be identified by measuring their behavior data and physiological data (HRV). Among them, HRV is the most important indicator to identify the three BPSDs.

3) A mobile robot was used for the first time as the implementation platform for home monitoring of BPSD. The feasibility has been demonstrated by the experiments in the virtual home environment. Non-constraint measurement of the HRV and walking speed can be realized effectively by the mobile robot with a fuzzy based following algorithm.

4) The monitoring of the various parameters will affect care giving and placement needs. It can help caregivers document daily activities, behavioral and vital signs, for assessing their healthy and live patterns, analyzing individual behavior changes for early detection of BPSD, and further exploring the cause of such changes in daily life. With the accumulated documents about BPSD identified and the situations likely to induce BPSD in home environment, it is possible to help decide suitable care plan for older persons living home. This can also help place the older persons in the relevant care setting.

### 5.4 Limitations

1) The data generated for training and testing the BPSD models exhibit a gap when compared to real-world data. However, this gap does not affect the validity of this study. The generated data are from real-world observation and investigation studies [9, 41–48], so that, it can reflect the real nature of BPSD, i.e., multivariate factors interacting with each other to affect one single or multiple BPSDs.

2) Only three BPSD were modelled, which may limit the application of the model. To identify more BPSDs, it is necessary to consider other factors and manifestations, such as the influence of living environment and cultural background. Though possibility to detect or observe those factors and manifestations within the current implementation platform needs to be considered.

3) An elderly individual with two or more BPSDs was not considered. It had been shown that an elderly individual may be affected with both agitation and apathy [72], apathy and depression [73], and depression and agitation [74] at the same time. The lack of consideration for elderly individuals with two or more BPSDs might be a reason resulted in incorrect judgments. In the future, we will consider the correlation between BPSDs and add the cases of “agitation & apathy, agitation & depression, depression & apathy” to the models. By investigating the common and specific factors between them, the identification of BPSD can be further improved.

4) Both implementation and validation of BePhyEn BPSD model were done in virtual environments. The uncertainty of home environment, BPSD patients, mobile robot and their interaction could not be taken into account, but also, the ethic and privacy issues and user acceptance issues could not be reflected, which might be influential factors when conducting real-world experiments.

5) Besides, The PsyCo BPSD model has been validated with the data generated but has not been validated in the virtual home environment.

## 6 Conclusion

Our study aims to develop a BPSD monitoring system that can detect and alert caregivers and care providers to BPSD. Based on a systematic analysis of the amount of data about BPSD manifestations in the literature, data sets for training and testing two BPSD models, the PsyCo BPSD model, the BePhyEn BPSD Model, were generated. The BePhyEn BPSD Model was further tested by a mobile robot monitoring platform implemented in a virtual environment, and three target following algorithms were compared. The results showed the proposed BePhyEn BPSD is feasible for BPSD identification in a virtual home environment.

## Figures and Tables

**Fig. 11:**
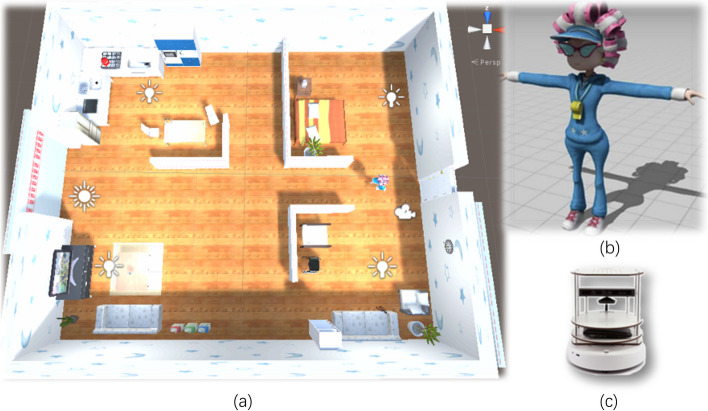
The construction of virtual home environment. **a** The construction of the home environment. **b** The construction of the elderly. **c** The construction of the robot

**Fig. 12:**
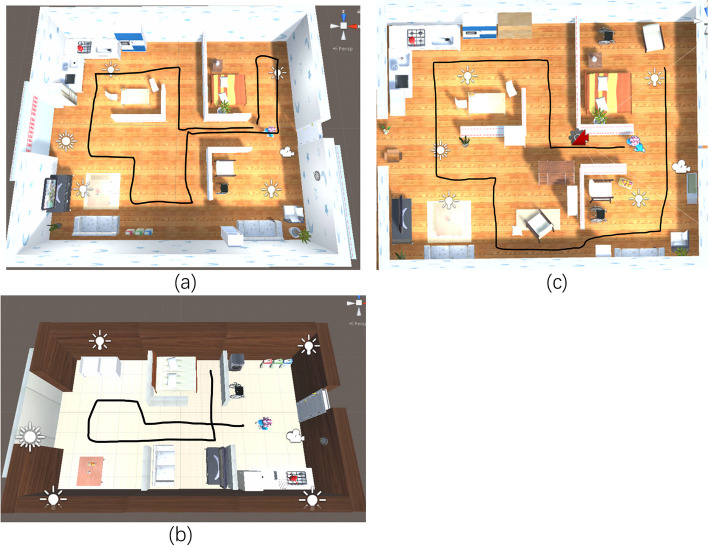
The fixed roadmap. **a** A large room has less obstructions. **b** A small room with more obstructions. **c** (a) A large room has more obstructions. Black line: the fixed roadmap was set

## Data Availability

The datasets used and/or analysed during the current study available from the corresponding author on reasonable request.

## Appendix

**1 Virtual home environment construction**

The virtual home environment was constructed as shown in Figure 11. Figure 11. (a) shows the construction of the virtual home environment, the room is provided by the SIGVerse project [75]. Figure 11. (b) shows the construction of elderly. After research and searching, there is a 3D model of "Sporty Granny" in Mixamo (https://www.mixamo.com/) that fits the requirements of this study. Figure 11. (c) shows the construction of the robot. The turtlrbot2 robot was selected, which was controlled by ROS (Robot Operating System, version: Kinetic). SIGVerse: The connection code from the SIGVerse project was used to create the connection between Unity and ROS.

**2 Different environment construction**

Rooms in home environment can be categorized into many types based on size, style, amount of occlusion, light intensity and so on. A total of three rooms were selected in this study to evaluate the environment in terms of room size and amount of occlusion, which are two important factors for target tracking algorithms. Room 1 is a typical home environment as referred to [75]. Room 2 is based on a typical Japanese single room as referred to [76]. Thus, they represent a relatively wide and small (about 160 and 70 square meters) typical home environment, respectively. Room 3 has the same room size, but with more furniture, thus, paired with Room 1, they represent a home environment with relatively more occlusions and less occlusions, respectively. Three rooms with corresponding fixed roadmaps as shown in Figure 12.(a)-(c) were built.
